# Epidemiologic and molecular risk factors for contralateral breast cancer among young women

**DOI:** 10.1038/sj.bjc.6601042

**Published:** 2003-07-29

**Authors:** C I Li, K E Malone, P L Porter, J R Daling

**Affiliations:** 1 Fred Hutchinson Cancer Research Center, Division of Public Health Sciences, 1100 Fairview Avenue North, MP-381, PO Box 19024, Seattle, WA 98109-1024, USA; 2 Fred Hutchinson Cancer Research Center, Division of Human Biology, 1100 Fairview Avenue North, MP-381, PO Box 19024, Seattle, WA 98109-1024, USA

**Keywords:** contralateral breast cancer, body mass index, c-erbB-2

## Abstract

Women diagnosed with a first breast cancer before the age of 45 years have a greater than 5.0-fold risk of developing a second primary contralateral breast cancer (CBC) than women in the general population have of developing a first breast cancer. Identifying epidemiologic or molecular factors that influence CBC risk could aid in the development of new strategies for the management of these patients. A total of 1285 participants in two case–control studies conducted in Seattle, Washington, who were 21–44 years of age when diagnosed with a first invasive breast carcinoma from 1983 to 1992, were followed through December 2001. Of them, 77 were diagnosed with CBC and 907 tumour tissues from first cancers were analysed. Women with body mass indices (BMIs) ⩾30 kg m^−2^ had a 2.6-fold greater risk (95% CI: 1.1–5.9) of CBC compared to women with BMIs ⩽19.9 kg m^−2^. Women whose first tumour was c-erbB-2 positive had a 1.7-fold (95% CI: 1.0–3.0) excess CBC risk. Body mass index and c-erbB-2 expression may be risk factors for CBC in young women. Further observational studies are needed to confirm these findings and to evaluate whether testing for c-erbB-2 in this population may help identify those at high risk for CBC.

The strongest identified risk factor for second primary contralateral breast cancer (CBC) is the age at diagnosis of a woman's first primary breast cancer (Chen *et al*, 1999). The younger a woman is diagnosed with an initial breast cancer, the greater her risk is of developing CBC. In a cohort study of 41 109 US women diagnosed with a first breast cancer, women diagnosed before the age of 45 years had a 5.4-fold greater risk of CBC than expected. Women of 45–54 and 55+ years of age had 3.4- and 1.3-fold greater risks of CBC, respectively (Harvey and Brinton, 1985). Smaller studies from England (Prior and Waterhouse, 1978), Sweden (Adami *et al*, 1985), Japan (Murakami *et al*, 1987), Germany (Brenner *et al*, 1993), and Slovenia (Volk and Pompe-Kirn, 1997) have reported similar results, finding that women <45 or <50 years of age have 3.0–9.9-fold greater risks of CBC than expected.

A family history of breast cancer and having a first breast tumour with a lobular histology have also been shown to increase CBC risk, while treatment of a first primary breast cancer with chemotherapy or tamoxifen has each been shown to decrease CBC risk (Chen *et al*, 1999). Although numerous studies have evaluated these and other risk factors for CBC, few have had sufficient power to evaluate epidemiologic or molecular risk factors among women diagnosed with a first breast cancer at a young age. Thus, little is known about what factors predispose young women diagnosed with a first breast cancer to develop CBC. To evaluate risk factors for CBC among women diagnosed with a first breast cancer at age younger than 45 years, we followed 1285 participants in a population-based case–control study, who were diagnosed with breast carcinoma from 1983 through 1992 in the Seattle-Puget Sound area of western Washington State for the occurrence of CBC.

## METHODS

### Case ascertainment and interviews

The 1488 breast carcinoma patients eligible for this study were women who had previously been interviewed in two population-based case–control studies. Women with *in situ* disease alone (*n*=203) were excluded from our study, and the remaining 1285 women with invasive breast cancer formed our cohort. Both methods of case–control studies were essentially the same and have been described previously (Daling *et al*, 1994; Brinton *et al*, 1995). The Cancer Surveillance System (CSS), a population-based cancer registry that is part of the Surveillance, Epidemiology, and End Results (SEER) Program of the National Cancer Institute, was used by both studies to identify women diagnosed with breast cancer who resided in King, Pierce, or Snohomish counties in western Washington state. The first study ascertained all incident cases of first *in situ* or invasive primary breast cancers among non-Hispanic white women who were diagnosed from 1 January 1983 through 30 April 1990 at 45 years of age or younger and who were born after 1944. Interviews were completed on 845 cases (83.3% of eligible cases). The second study ascertained all incident first *in situ* and invasive primary breast cancer cases of any race who were diagnosed from 1 May 1990 through 31 December 1992 at age less than 45 years. Interviews were completed on 643 (83.9%) of all eligible cases.

Participants in both studies were interviewed in their homes by a trained interviewer. Respondents were asked about their reproductive history, family history of breast cancer, and their body size history. Weight 1 year prior to diagnosis was used to determine body mass index (BMI) for this study. In our analyses, BMI was grouped using Bray's criteria (Bray, 1987) (where women with a BMI ⩽19.9 kg m^−2^ are considered underweight, 20.0–24.9 kg m^−2^ as normal weight, 25.0–29.9 kg m^−2^ as overweight, and ⩾30.0 kg m^−2^ as obese) and into quartiles. Information on ever use of treatments for the subjects' first breast cancer, including chemotherapy, radiation, and tamoxifen, was subsequently obtained from three sources: (1) medical record abstractions, (2) telephone and written contacts with subjects or their proxies, and (3) CSS. Informed consent was obtained from all subjects prior to participation.

### Follow-up for CBC

The source of information on occurrence of CBC in the cohort was CSS, which follows all cancer cases for vital status and the diagnosis of subsequent primary cancers. The length of follow-up time for cohort members was measured as the time from 6 months after the diagnosis of their first breast cancer to diagnosis of CBC; date of last follow-up; death; or the end of the study period (December 2001), whichever occurred first. Women were considered to have developed CBC if their second breast tumour was: (1) diagnosed greater than 6 months after their first breast cancer was diagnosed; (2) was diagnosed in the opposite breast; and (3) was invasive. All participants had a follow-up time of 6 months or greater. The registry annually contacts the hospital tumour registrar for information on the disease status and vital status of each patient. The tumour registrar then contacts the physician following each patient for an updated determination of vital status. If there is no physician who has had sufficiently recent contact with the patient, then the registrar sends a letter to the patient. This follow-up for both disease status and vital status is performed for cases, regardless of whether or not they currently reside in the CSS catchment area. In addition, passive surveillance through routine computer linkage of patients with Washington State death certificates, the National Death Index, and Health Care Finance Administration tapes is conducted. The status of greater than 85% of the cohort was known within 2 years of December 2001.

### Tissue collection, pathology review, and testing for molecular markers

Data on tumour stage, histology, and size were obtained from CSS. Tumour specimens were requested from hospital and commercial pathology laboratories. We were unable to obtain permission to request tumour tissue from 4.1% of the women. In addition, 25.3% of the tumour blocks were not available or had been discarded by the pathology laboratories.

Tumour tissue sufficient for immunoperoxidase assays was available for 907 (70.6%) of the study participants. The study pathologist (Peggy L Porter, MD) conducted a complete histopathologic review for all the tumours collected. The CBC status and clinical and personal characteristics of the women were unknown at the time of review. Expression of oestrogen receptor (ER), progesterone receptor (PR), p53 tumour suppression gene protein, Ki-67 proliferation-related antigen, c-erbB-2 oncogene protein, apoptosis regulatory protein bcl-2, and cell cycle proteins cyclin E and p27 were evaluated on sections from a single tumour block as described previously (Porter *et al*, 1997, 1999).

Antibodies were scored using a subjective interpretation of staining intensity and/or the percentage of tumour cells positive. Categories of intensity and/or percent cells positive were collapsed into positive/high or negative/low categories as follows: for ER and PR, any nuclear staining was considered positive; the percentage of Ki-67 positive tumour cells was averaged over four high-powered fields and converted to quartiles; the nuclear staining of >10% tumour cells for p53 was considered positive; a distinct membranous staining pattern of 1–3+ was considered positive for c-erbB-2. The antibody staining for c-erbB-2 was carried out using the AO485 antibody from DAKO and was initiated prior to the acceptance of the DAKO's Herceptest kit as the FDA-approved technique for the evaluation of c-erbB-2 expression. The clinical use of this marker to determine therapy was not the objective of this study and as such, we assigned tumours that demonstrated a clear positive membranous stain over that of normal breast epithelium to the positive category. The intensity of bcl-2 stains was categorised as negative, low, intermediate, and high. Expression of cyclin E and p27 was scored as a value from 1 (negative) to 7 (highest intensity). Low intensity included all values of 1–4 and high intensity included values from 5 to 7.

Bivariate flow cytometric analysis of DNA content and S-phase fraction was completed for 694 (59%) of the tumours, as described previously (Glogovac *et al*, 1996).

### Statistical analysis

Associations between different epidemiologic and molecular risk factors for CBC were estimated using the Cox proportional hazard model (Cox, 1972). Using Stata 7.0 (College Station, TX for Windows (Stata Corporation, Chicago, IL, USA) statistical software, Cox regression was performed to compute hazard ratios and 95% confidence intervals (CI) and to evaluate the effects of confounding and modifying factors. Systematic evaluation for confounding by other potential confounders, including stage, histologic type, and other treatments, was performed. All analyses were adjusted for age and year at diagnosis, American Joint Committee on Cancer (AJCC) stage, chemotherapy, and the original study in which the subjects had participated.

## RESULTS

The mean age at diagnosis of first primary breast cancer for the entire cohort was 37. 7 years, and the mean follow-up time was 9.0 years (range: 0.5–18.1 years) (Table 1

**Table 1:** Characteristics of a cohort of 1285 women diagnosed with a first primary breast cancer between 1 January 1983 and 31 December 1992

	**Entire cohort (n=1285)**		**Cohort with tumour marker data (n=907)**	
**Characteristics**	**Average %**	**Missing data (%)**	**Average %**	**Missing data (%)**
Age (years) (s.d.)	37.7 (4.3)	0.0	38.0 (4.2)	0.0
Follow-up (years) (s.d.)	9.0 (3.8)	0.0	8.5 (3.5)	0.0
Age at first birth (years) (s.d.)	24.3 (5.1)	0.3	24.7 (5.2)	0.1
Body mass index (kg m^−2^) (s.d.)	23.9 (5.0)	1.4	24.1 (5.0)	1.7
				
*Family history (%)*		1.8		1.8
None	63.2		64.1	
First-degree relative affected	15.6		15.2	
Second-degree relative affected	21.2		20.8	
				
*AJCC stage (%)*		1.9		0.6
I	37.5		37.5	
II	51.0		51.3	
III	8.7		9.0	
IV	2.7		2.2	
Lobular histology (%)	3.4	0.0	3.6	0.0
Tumour size (cm) (s.d.)	1.7 (3.4)	20.7	1.5 (3.3)	0.0
				
*Breast cancer treatments received (%)*				
Chemotherapy	71.5	0.2	74.4	0.0
Radiation	56.8	0.2	56.7	0.1
Tamoxifen	38.3	8.7	41.5	4.5

). The average age at first live birth was 24.3 years and the mean BMI was 23.9 kg m^−2^. Additionally, 15.6% of the cohort had a first-degree family history of breast cancer and 21.2% had a second-degree family history. The majority of women were diagnosed with either stage I or II breast cancer, the mean tumour size was 1.7 cm, and only 3.4% of the women had lobular carcinoma. In all, 71.5% of the cohort had their first breast cancer treated with chemotherapy, 56.8% received radiation therapy, and 38.3% ever used tamoxifen. In general, the cohort of women, whose tumour tissue was evaluated for molecular markers, did not differ from the entire cohort with respect to these characteristics.

Multivariable-adjusted estimates of risk for CBC in relation to various patient characteristics are presented in Table 2

**Table 2:** Risk of CBC by patient characteristics at the time of first cancer diagnosis

	**Number of**				
	**Subjects at risk**	**P-years**	**Number of CBC cases**		
**Patient Characteristics**	***n*=1285**	**at risk**	***n*=77**	**HR**	**95% CI**
*Age at diagnosis (years)*					
⩽29	65	592	7	2.8	1.1, 6.9
30–34	201	1848	17	2.1	1.1, 4.4
35–39	477	4375	36	1.9	1.1, 3.5
40+	515	4444	17	1.0	Ref.
					
*Age at menarche (years)*					
8–12	665	6020	35	1.0	Ref.
13–15	550	4865	40	1.5	0.9, 2.3
16+	43	374	2	0.9	0.2, 3.7
					
*Gravidity*					
Never pregnant	204	1857	16	1.0	Ref.
Ever pregnant	1,053	9391	61	0.9	0.5, 1.6
					
*Age at first live birth (years)*					
<20	177	1626	8	1.0	Ref.
20–29	590	5272	39	1.5	0.7, 3.2
30+	161	1321	9	1.5	0.6, 4.0
					
*Number of live births*					
None	331	3041	21	1.0	Ref.
1–3	856	7566	50	1.2	0.7, 2.0
4+	70	641	6	1.7	0.7, 4.4
					
*Oral contraceptive use (years)*					
None	282	2451	16	1.0	Ref.
<10	807	7319	48	1.0	0.6, 1.8
10+	169	1489	13	1.3	0.6, 2.7
					
*BMI, using Bray's criteria (kg m^−2^)*					
⩽19.9	212	2104	11	1.0	Ref.
20.0–24.9	669	6077	43	1.6	0.8, 3.1
25.0–29.9	205	1743	11	1.5	0.6, 3.6
30.0+	154	1200	12	2.6	1.1, 5.9
					
*BMI, quartiles (kg m^−2^)*					
⩽20.6	309	3008	16	1.0	Ref.
20.7–22.4	312	2911	18	1.2	0.6, 2.4
22.5–25.8	311	2703	26	2.1	1.1, 3.9
25.9+	316	2550	17	1.6	0.8, 3.2
					
*Weight, quartiles (lb)*					
⩽123	331	3178	14	1.0	Ref.
124–135	320	3003	18	1.5	0.7, 3.0
136–155	311	2656	27	2.8	1.4, 5.3
156+	294	2405	18	2.2	1.1, 4.4
					
*Average number of drinks per week*					
None/<1	507	4421	31	1.0	Ref.
1–3	297	2689	17	0.9	0.5, 1.6
3+	452	4134	29	0.9	0.5, 1.5
					
*Family history of breast cancer*					
None	778	6838	40	1.0	Ref.
First degree	193	1850	17	1.5	0.9, 2.7
Second degree	264	2395	18	1.3	0.8, 2.3

HRs for age at diagnosis are adjusted for year at diagnosis, AJCC stage, chemotherapy, and the study that the subjects participated in. All other HRs are adjusted for age and year at diagnosis, AJCC stage, chemotherapy, and the study that the subjects participated in. P-years=patient-years; HR=hazard ratio; AJCC=American Joint Committee on cancer; Ref.=reference.

. Women diagnosed with their first breast cancer when they were ⩽29, 30–34, and 35–39 years of age had 2.8 (95% CI: 1.1–6.9), 2.1 (95% CI: 1.1–4.4), and 1.9-fold (95% CI: 1.1–3.5) greater risks of CBC compared to women diagnosed at 40–44 years of age, respectively. Women with first live births after the age of 20 years and with a greater number of live births also appeared to have greater risks of CBC, but these increases were within the limits of chance. Using Bray's criteria (Bray, 1987), women who were obese (BMI ⩾30.0 kg m^−2^) when diagnosed with their first breast cancer also had an increased risk of CBC compared to women with a BMI ⩽19.9 kg m^−2^. When assessed as quartiles, women in the upper two quartiles of BMI had a greater risk of CBC compared to women in the lowest quartile (although the increase in risk for those in the highest quartile was within the limits of chance). With respect to weight, women who were in the upper two quartiles of weight had a greater risk of CBC compared to women in the lowest quartile. Alcohol use was not associated with an increased risk of CBC. Finally, a first-degree family history increased CBC risk 1.5-fold, although this increase was within the limits of chance.

Neither stage, histology, nor tumour size of the first tumour appeared to alter CBC risk (Table 3

**Table 3:** Risk of CBC by tumour characteristics of first cancer diagnosis

	**Number of**				
	**Subjects at risk**	**P-years**	**Number of CBC cases**		
**Tumour Characteristics**	***n*=1285**	**at risk**	***n*=77**	**HR**	**95% CI**
*AJCC stage*					
I	473	4587	31	1.0	Ref.
II	641	5702	42	1.0	0.6, 1.6
III	110	842	3	0.5	0.1, 1.7
IV	34	128	1	1.1	0.1, 8.1
					
*Histology*					
Ductal	922	8370	59	1.0	Ref.
Medullary	44	395	2	0.9	0.2, 3.6
Lobular	52	492	3	0.7	0.2, 2.3
Other	265	2248	13	0.9	0.5, 1.6
					
*Size (cm)*					
⩽2.0	653	6167	39	1.0	Ref.
>2.0–5.0	498	4271	34	1.2	0.7, 1.9
>5.0	102	803	4	0.7	0.3, 2.1

HRs adjusted for age and year at diagnosis, AJCC stage, chemotherapy, and the study subjects participated in. P-years=patient-years; HR=hazard ratio; AJCC=American Joint Committee on cancer; Ref.=reference.

). With respect to the tumour markers expressed by these first tumours, women whose first tumour was c-erbB-2 positive had a 1.7-fold (95% CI: 1.0–3.0) increased risk of CBC (Table 4

**Table 4:** Risk of CBC by tumour markers expressed by the first breast cancer

	**Number of**				
	**Subjects at risk**	**P-years**	**Number of CBC cases**		
**Tumour marker**	***n*=907**	**at risk**	***n*=51**	**HR**	**95% CI**
*ER*					
Positive	533	4612	31	1.0	Ref.
Negative	364	3020	20	0.8	0.5, 1.5
					
*PR*					
Positive	541	4691	30	1.0	Ref.
Negative	354	2696	21	1.0	0.6, 1.8
					
*Bcl-2*					
Negative	331	2730	15	1.0	Ref.
Low	183	1555	11	1.4	0.6, 3.0
Intermediate	175	1532	12	1.4	0.7, 3.1
High	201	1786	12	1.4	0.6, 2.9
					
*Cyclin E*					
High	672	5665	37	1.0	Ref.
Low	219	1750	14	1.1	0.6, 2.0
					
*Ki-67*					
0–24%	537	4622	27	1.0	Ref.
25–100%	354	2965	24	1.3	0.7, 2.3
					
*c-erbB-2*					
Negative	490	4375	23	1.0	Ref.
Positive	407	3256	27	1.7	1.0, 3.0
					
*p27*					
Intermediate/high	465	4098	28	1.0	Ref.
Low	426	3501	23	0.8	0.5, 1.5
					
*p53*					
Negative	538	4713	32	1.0	Ref.
Positive	360	2920	19	0.9	0.5, 1.6
					
*S phase*					
Low	323	2825	20	1.0	Ref.
High	327	2794	20	0.9	0.5, 1.8

HRs adjusted for age and year at diagnosis, AJCC stage, chemotherapy, and the study subjects participated in. P-years=patient-years; HR=hazard ratio; ER: oestrogen receptor; PR=progesterone receptor; Ref.=reference.

). However, other markers, including ER, PR, bcl-2, cyclin E, Ki-67, p27, p53, and S phase were not associated with CBC risk.

## DISCUSSION

Although numerous studies have assessed risk factors for CBC, few have assessed CBC risk factors among women diagnosed with their first breast cancer before the age of 45 years. Since the risk of CBC increases the younger a woman is diagnosed with a first breast cancer, as this and other studies suggest, identification of factors that contributes to their risk is important.

In this study, we observed that both obesity (defined as a BMI ⩾30.0 kg m^−2^) and a total body weight in the upper two quartiles at the time women were diagnosed with their first breast were associated with increases in CBC risk. The relation of BMI to breast cancer risk and mortality is complex. A high BMI is protective of premenopausal breast cancer, but increases a woman's risk of postmenopausal breast cancer. However, BMI is also positively correlated with risk of mortality among both premenopausal (Greenberg *et al*, 1985; Holmberg *et al*, 1994; Lethaby *et al*, 1996; Daling *et al*, 2001) and postmenopausal (Goodwin and Boyd, 1990) women with breast cancer. The relation between BMI and CBC risk is less clear, although one study reports that CBC risk increases as BMI increases (Storm *et al*, 1992) and others report that BMI does not influence CBC risk (Hislop *et al*, 1984; Horn and Thompson, 1988; Bernstein *et al*, 1992). However, none of these studies have assessed women diagnosed with their first breast cancer before the age of 45 years separately. While our finding is novel, it needs to be confirmed by others, and further research is required to identify mechanisms underlying an association between body weight and CBC.

There are limitations of our data concerning body weight and BMI as the data on all patient characteristics were based on self-reports. This may have been a particular problem for our analyses of weight and BMI (calculated from self-reported heights and weights), since participants in research studies may underestimate their weight. This issue has been addressed to some extent since women in the second case–control study who formed our cohort were weighed and measured at the time of their interview. It has been shown that the correlation between self-reported weight 1 year before diagnosis and weight measured at the interview for these women was *R*=0.89 (Daling *et al*, 2001), suggesting that self-reported weight is reliable among women in our study.

With respect to molecular markers that are related to CBC risk, we found that expression of c-erbB-2 was associated with an increased CBC risk in our cohort. Given that c-erbB-2 is a potent oncogene and a strong predictor of prognosis, this finding is not surprising, although it needs to be verified by others. While we did not find that any of the other markers we evaluated significantly altered CBC risk, this study was limited by a relatively small number of CBC cases and thus may have lacked sufficient power to detect these associations. Furthermore, this study is largely hypothesis generating and therefore these associations need to be viewed tentatively. Additional insight into the molecular aetiology of CBC is needed and could be gained by the assessment of these and other markers in the contralateral tumours themselves and by comparing them to those expressed by first primary breast tumours.

In summary, we present the first data from a large group of young women with breast cancer and find that high BMI at the time of a first breast cancer diagnosis and c-erbB-2 expression by this tumour, both appear to be important risk factors for CBC. If these associations are borne out in future studies, the subgroups of women who either have a high BMI or whose first tumours overexpress c-erbB-2 may warrant closer screening for CBC. Additionally, it will be important to establish whether interventions such as treatment with c-erbB-2-targeted therapies, such as trastuzumab, after diagnosis with breast cancer alter the incidence of CBC in young women.
